# Supplementary material for: Mechanisms governing the pioneering and redistribution capabilities of the non-classical pioneer PU.1
Source: Nat Commun. 2020 Jan 21;11:402. doi: 10.1038/s41467-019-13960-2 (PMC6972792; doi:10.1038/s41467-019-13960-2)
Supplement: Supplementary file 7 — Source data [file 41467_2019_13960_MOESM7_ESM.zip › Source_Data/Figure6/Figure6B_MotifScanOutput/ETS1_PU.1/homerResults/motif34.similar.html]

motif34

## Information for motif34

C
T
A
G
T
G
A
C
T
C
A
G
T
A
G
C
G
C
T
A
C
A
G
T
A
T
C
G
A
T
G
C
A
C
T
G
G
A
T
C
T
C
G
A
  
Reverse Opposite:  

A
G
C
T
C
T
A
G
T
G
A
C
A
T
C
G
A
T
G
C
G
T
C
A
C
G
A
T
A
T
C
G
A
G
T
C
A
C
T
G
G
A
T
C
  

|  |  |
| --- | --- |
| p-value: | 1e-44 |
| log p-value: | -1.035e+02 |
| Information Content per bp: | 1.729 |
| Number of Target Sequences with motif | 416.0 |
| Percentage of Target Sequences with motif | 2.63% |
| Number of Background Sequences with motif | 290.9 |
| Percentage of Background Sequences with motif | 0.91% |
| Average Position of motif in Targets | 99.6 +/- 55.7bp |
| Average Position of motif in Background | 91.2 +/- 67.4bp |
| Strand Bias (log2 ratio + to - strand density) | 0.0 |
| Multiplicity (# of sites on avg that occur together) | 1.16 |
| Motif File: | file (matrix) reverse opposite |

### Similar de novo motifs found

|  |  |  |  |  |  |  |  |
| --- | --- | --- | --- | --- | --- | --- | --- |
| Rank | Match Score | Redundant Motif | P-value | log P-value | % of Targets | % of Background | Motif file |
| 1 | 0.926 | T G A C T A C G A T G C G T C A C A G T T A C G T G A C A C T G G A T C C G T A | 1e-41 | -94.964302 | 2.36% | 0.80% | motif file (matrix) |
| 2 | 0.991 | C T A G T G A C T C A G A T G C G T C A A C G T A T C G A G T C A C T G A G T C T C G A A T C G | 1e-37 | -86.316487 | 2.62% | 1.02% | motif file (matrix) |
| 3 | 0.976 | A C T G C T A G A G T C A C T G A G T C C G T A A C G T A C T G A G T C A C T G A G T C T G C A T A G C | 1e-36 | -83.959007 | 2.51% | 0.96% | motif file (matrix) |
| 4 | 0.941 | A C G T A C T G A G T C A C T G A G T C G T C A A C G T A C T G A G T C A C T G A G T C C T G A T A C G C G T A | 1e-35 | -82.274239 | 2.39% | 0.90% | motif file (matrix) |
| 5 | 0.934 | C T A G G T A C A C T G G T A C G C T A C A G T A C T G T A G C C A T G | 1e-35 | -80.656190 | 2.19% | 0.79% | motif file (matrix) |
